# Long-term efficacy and safety of CT-P6 versus trastuzumab in patients with HER2-positive early breast cancer: final results from a randomized phase III trial

**DOI:** 10.1007/s10549-021-06240-5

**Published:** 2021-06-20

**Authors:** Justin Stebbing, Yauheni V. Baranau, Valery Baryash, Alexey Manikhas, Vladimir Moiseyenko, Giorgi Dzagnidze, Edvard Zhavrid, Dmytro Boliukh, Joanna Pikiel, Alexandru E. Eniu, Rubi K. Li, Beatrice Tiangco, Sang Joon Lee, Sunghyun Kim

**Affiliations:** 1 Division of Cancer, Imperial Centre for Translational and Experimental Medicine, London, UK; 2 Imperial College Healthcare NHS Trust, Charing Cross Hospital, London, UK; 3 Department of Oncology, Belarusian State Medical University, Minsk, Belarus; 4 City Clinical Oncology Dispensary, Saint Petersburg, Russian Federation; 5 Clinical Research Center of Specialised Types of Care (Oncology), GBUZ Saint Petersburg, Saint Petersburg, Russian Federation; 6 S. Khechinashvili University Clinic, Ltd, Tbilisi, Georgia; 7 N.N. Alexandrov National Cancer Centre of Belarus, Minsk Region, Belarus; 8 Vinnytsya Regional Clinical Oncology Dispensary, Vinnytsia, Ukraine; 9 Wojewodzkie Centrum Onkologii, Gdańsk, Poland; 10 Hôpital Riviera-Chablais, Vaud-Valais, Rennaz, Switzerland; 11 St Luke’s Medical Center, Quezon City, Philippines; 12 The Medical City, Ortigas Avenue, Pasig City, Philippines; 13 Celltrion, Inc., Incheon, Republic of Korea

**Keywords:** Adjuvant therapy, Biosimilar, CT-P6, HER2-positive early breast cancer, Neoadjuvant therapy, Trastuzumab

## Abstract

**Purpose:**

Equivalent efficacy was demonstrated for the biosimilar CT-P6 and trastuzumab following neoadjuvant therapy for patients with human epidermal growth factor receptor-2 (HER2)-positive early breast cancer. Following adjuvant treatment, efficacy and safety were comparable between treatments. We report updated safety and efficacy data after up to 3 years’ follow-up.

**Methods:**

Following neoadjuvant chemotherapy with CT-P6/trastuzumab, patients underwent surgery and continued receiving adjuvant CT-P6/trastuzumab. The primary endpoint (previously reported) was pathological complete response. Time-to-event analyses (disease-free survival [DFS], progression-free survival [PFS], and overall survival [OS]), study drug-related and cardiac adverse events, and immunogenicity were assessed during post-treatment follow-up.

**Results:**

Most patients entered the follow-up period (CT-P6: 259 [95.6%]; trastuzumab: 269 [96.8%]). After a median follow-up of 38.7 (CT-P6) and 39.6 (trastuzumab) months, medians were not reached for time-to-event parameters; estimated hazard ratios (HRs) and 3-year survival rates were similar between groups. Estimated HRs (95% confidence intervals) for CT-P6 versus trastuzumab were 1.23 (0.78–1.93) for DFS, 1.31 (0.86–2.01) for PFS, and 1.10 (0.57–2.13) for OS (intention-to-treat population). Safety findings were comparable between groups for the overall study and follow-up period, including study drug-related cardiac disorders (CT-P6: 22 [8.1%] patients; trastuzumab: 24 [8.6%] patients [overall]) and decreases in left ventricular ejection fraction. Immunogenicity was similar between groups.

**Conclusion:**

The similarity of the time-to-event analyses between CT-P6 and trastuzumab supports the equivalence in terms of efficacy established for the primary endpoint. CT-P6 was well tolerated, with comparable safety and immunogenicity to trastuzumab.

ClinicalTrials.gov: NCT02162667 (registered June 13, 2014)

**Supplementary Information:**

The online version contains supplementary material available at 10.1007/s10549-021-06240-5.

## Introduction

Trastuzumab plays a key role in the treatment of patients with human epidermal growth factor receptor-2 (HER2)-positive early breast cancer. For example, adjuvant chemotherapy with trastuzumab is highlighted in the National Comprehensive Cancer Center (NCCN) guidelines for the treatment of HER2-positive breast cancer [1], while the European Society for Medical Oncology clinical practice guidelines recognize that “(neo)adjuvant trastuzumab is highly effective and should be given to all HER2-positive early breast cancer patients who do not have contraindications for its use,” with limited exceptions [2]. NCCN guidelines note that a trastuzumab biosimilar licensed by the US Food and Drug Administration (FDA) is “an appropriate substitute” for the reference product [1]. One such trastuzumab biosimilar is CT-P6, which is licensed by both the FDA and European Medicines Agency for the same indications for HER2-overexpressing cancers as the reference product [3–6]. The comparability of CT-P6 and trastuzumab has been demonstrated in terms of structural, physicochemical, and biological activity [7], as well as for the in vitro mechanism of action [8]. In addition, the pharmacokinetic equivalence and similar safety of CT-P6 and trastuzumab have been demonstrated in a phase I, single-dose study in healthy adult males [9].

The current phase III study compared the efficacy, safety, immunogenicity, pharmacokinetics, and pharmacodynamics of CT-P6 and trastuzumab, administered as both neoadjuvant and adjuvant therapy for patients with operable HER2-positive early breast cancer [10, 11]. Equivalent efficacy was demonstrated between CT-P6 and trastuzumab in terms of pathological complete response rate following neoadjuvant treatment [10]. Other assessments and safety outcomes were comparable between groups during the neoadjuvant period [10]. Outcomes of adjuvant therapy with CT-P6, in terms of preventing disease progression or recurrence and safety profile, were also comparable to those with trastuzumab treatment [11]. This article reports long-term survival outcomes and safety findings, including cardiac toxicity, after up to 3 years’ follow-up during the post-treatment period of the study.

## Methods

### Study design and participants

Full details of this randomized, double-blind, active-controlled, phase III study (NCT02162667) have been published [10, 11]. Patients were recruited from 112 centers in 23 countries. Patients received neoadjuvant treatment with eight 3-week cycles of CT-P6 (Herzuma^®^; Celltrion, Inc., Incheon, Republic of Korea) or trastuzumab (Herceptin^®^; Genentech, San Francisco, CA, USA), both administered at a loading dose of 8 mg/kg on day 1 of cycle 1 and then at 6 mg/kg on day 1 of cycles 2–8, with docetaxel and fluorouracil, epirubicin, and cyclophosphamide, followed by surgery (Fig. S1 in Online Resource 1). Patients then received ≤ 10 cycles of adjuvant CT-P6 or trastuzumab (6 mg/kg administered every 3 weeks, per original randomization) before entering a post-treatment follow-up period, which extended until 3 years from the day of enrollment of the last patient.

Eligible patients were women aged ≥ 18 years with histologically confirmed, newly diagnosed HER2-positive breast cancer of clinical stage I–IIIa per American Joint Committee on Cancer Breast Cancer Staging, Seventh Edition [10]. Patients were required to have a left ventricular ejection fraction (LVEF) of ≥ 55% at baseline [10]. Key exclusion criteria included bilateral breast cancer, prior breast cancer treatment, and prior anthracycline treatment [10].

### Endpoints and assessments

The primary efficacy endpoint was pathological complete response rate analyzed in the per-protocol population, as previously described [10]. Secondary efficacy endpoints assessed during the study period were disease-free survival (DFS), defined as the interval between the date of breast surgery and disease progression, recurrence, or death from any cause; progression-free survival (PFS), defined as the interval between randomization and disease progression, recurrence, or death from any cause; and overall survival (OS), defined as the interval between randomization and death from any cause. DFS and PFS endpoints used disease status assessment by mammogram, physical examination, other radiological methods on the tumor site, or clinical symptoms.

Assessments conducted during the post-treatment follow-up period included physical examination of the tumor site and tumor response evaluation every 3 months (until disease progression or recurrence), mammogram every year (for patients with non-measurable lesions eligible for assessment), and chest X-ray every 6 months (at the investigator’s discretion). Survival status and any salvage therapy were recorded every 3 months.

Adverse events (AEs) were recorded until 30 days after the last dose of study drug. AEs were coded using the Medical Dictionary for Regulatory Activities, Version 18.1. Between 30 days after last study drug administration and the end of the study, only study drug-related AEs and cardiac AEs were reported. For cardiac monitoring, 12-lead electrocardiogram, echocardiogram, or multiple-gated acquisition scan (to determine LVEF) and New York Heart Association class evaluation were conducted every 6 months for up to 2 years (for a maximum of four times), with additional evaluations conducted if clinically indicated during the post-treatment follow-up.

Blood samples for immunogenicity testing were obtained every 3 months for up to 1 year (to a maximum of four samples). Antidrug antibodies (ADAs) against CT-P6 or trastuzumab were detected in serum samples using an electrochemiluminescence assay based on the Meso Scale Discovery platform (Meso Scale Discovery, Rockville, MD, USA), which was validated as part of the CT-P6 development process. Samples positive for ADAs on screening were confirmed as positive using the same assay platform in a competitive inhibition format. ADA-positive samples were further characterized using a cell-based neutralization assay.

### Statistical analyses

Sample size was determined as previously described [10]. Hazard ratios (HRs) and 95% confidence intervals (CIs) comparing CT-P6 with trastuzumab for time-to-event analyses were estimated using an adjusted stratified Cox regression model with disease stage (stage I or II vs stage IIIa or higher), estrogen receptor status (positive vs negative), progesterone receptor status (positive vs negative), and region (Europe, the Middle East, and Africa vs Asia vs America) as stratification factors. The Kaplan–Meier method was used to analyze median survival times and 3-year survival rates. Time-to-event analyses were conducted in the intention-to-treat (ITT) and per-protocol populations. The ITT population comprised all patients randomized to study drug, regardless of whether a dose of study drug was received. The per-protocol population comprised all patients included in the ITT population, other than any patients excluded because of major protocol deviations. Safety analyses were conducted in the safety population, comprising all patients randomized to study drug who had received at least 1 (full or partial) dose.

## Results

### Patient disposition

**Fig. 1:**
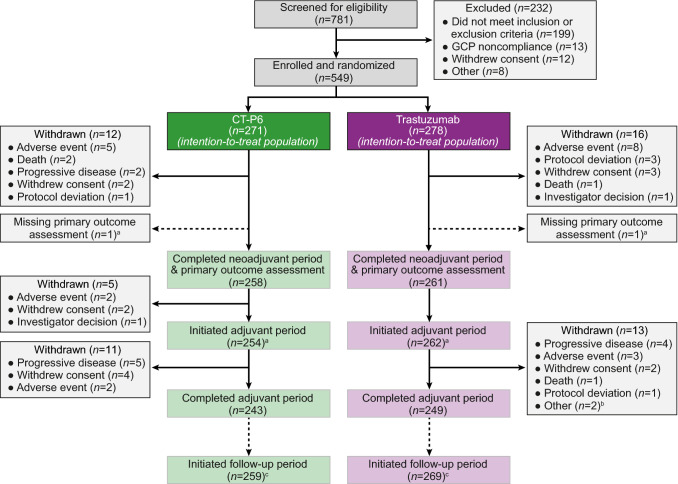
Patient disposition. ^a^Following completion of the neoadjuvant period and surgery, 1 patient in each treatment group initiated the adjuvant period but did not complete the primary outcome assessment owing to lost pathology samples. ^b^Relocation (*n* = 1) and being unable to visit the study center within the visit window (*n* = 1). ^c^Patients entered the post-treatment follow-up period regardless of completion of treatment, provided they did not withdraw consent. *GCP* Good Clinical Practice. Figure adapted from Ref. [11], as permitted under the terms of the Creative Commons Attribution 4.0 International License (http://creativecommons.org/licenses/by/4.0/)

Patients were randomized between August 7, 2014 and October 20, 2015; the date of the last patient’s last follow-up was October 23, 2018. Figure 1 shows the patient disposition. Most patients (CT-P6: 259 [95.6%]; trastuzumab: 269 [96.8%]) entered the follow-up period. As previously described, baseline patient demographics and disease characteristics were similar between groups [10].

### Time-to-event analyses

**Fig. 2:**
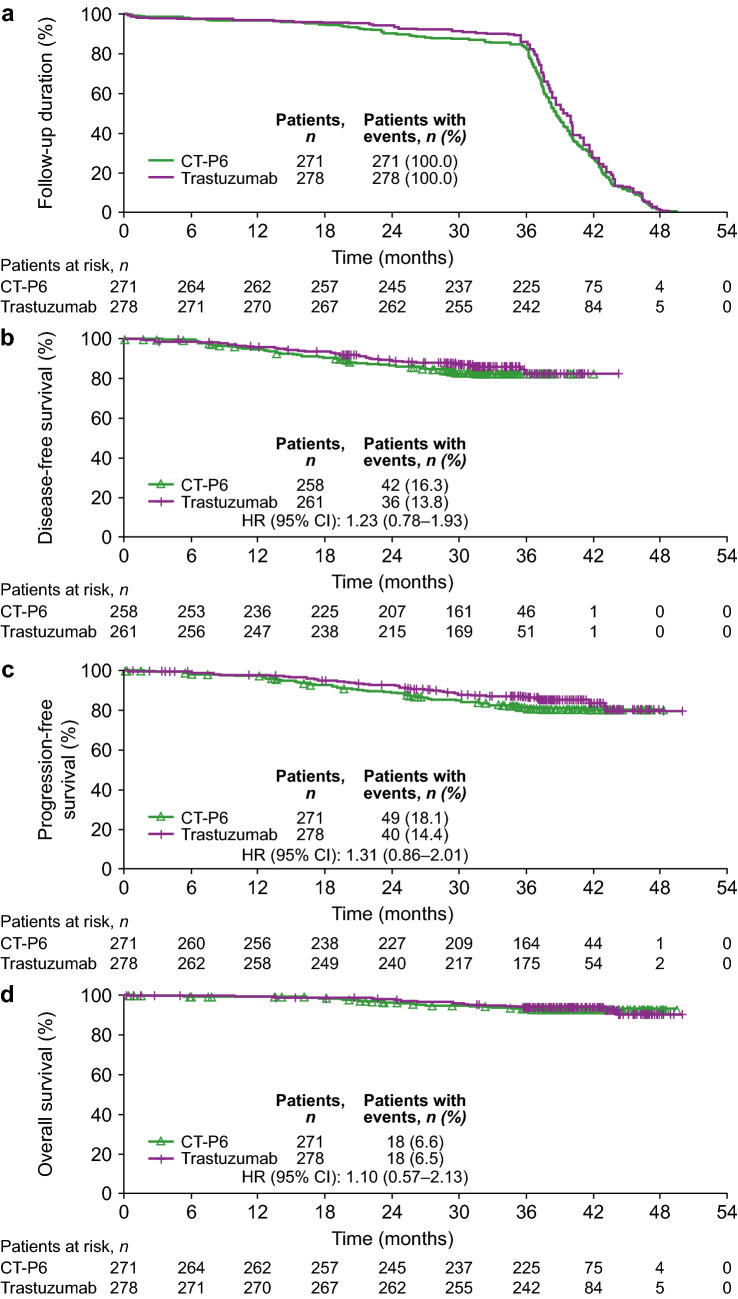
Kaplan–Meier plots for time-to-event analyses (intention-to-treat population). **a** Follow-up duration, **b** disease-free survival, **c** progression-free survival, **d** overall survival. *CI* confidence interval, *HR* hazard ratio

In the ITT population, the median (95% CI) follow-up duration was similar between the CT-P6 and trastuzumab groups (38.7 [38.1–39.4] and 39.6 [38.9–40.1] months, respectively) (Fig. 2a).

Median DFS was not yet reached in either group in the ITT population due to an insufficient number of events (Fig. 2b). Events were most commonly progressive disease/recurrence (CT-P6: 41 [15.9%] patients; trastuzumab: 33 [12.6%] patients). In both treatment groups, most progressive disease/recurrence events were distant rather than locoregional (Table S1 in Online Resource 1). Deaths accounted for the remaining events (CT-P6: 1 [0.4%] patients; trastuzumab: 3 [1.1%] patients). The estimated HR was 1.23 (95% CI 0.78–1.93) (Fig. 2b). DFS was similar between groups in terms of the 3-year rate (CT-P6: 0.83 [95% CI 0.77–0.87]; trastuzumab: 0.83 [95% CI 0.76–0.88]).

Median PFS was not yet reached in either group in the ITT population due to an insufficient number of events (Fig. 2c). Events were most commonly progressive disease/recurrence (CT-P6: 45 [16.6%] patients; trastuzumab: 35 [12.6%] patients); deaths accounted for the remaining events (CT-P6: 4 [1.5%] patients; trastuzumab: 5 [1.8%] patients). One death in each treatment group was breast cancer-related. The estimated HR was 1.31 (95% CI 0.86–2.01) (Fig. 2c), and PFS was similar between groups in terms of the 3-year rate (CT-P6: 0.81 [95% CI 0.76–0.85]; trastuzumab: 0.87 [95% CI 0.82–0.90]).

Median OS was not yet reached in either group in the ITT population due to an insufficient number of events (Fig. 2d); however, the estimated HR was 1.10 (95% CI 0.57–2.13) (Fig. 2d). OS was similar between groups in terms of the 3-year rate (CT-P6: 0.93 [95% CI 0.90–0.96]; trastuzumab: 0.94 [95% CI 0.90–0.96]).

Findings for time-to-event analyses conducted in the per-protocol population were similar to results in the ITT population (Fig. S2 in Online Resource 1).

### Safety and immunogenicity

During the overall study, the proportions of patients experiencing treatment-emergent AEs (TEAEs) were similar between groups (Table 1). The most frequently reported TEAEs in the CT-P6 group were alopecia (196 [72.3%] patients), nausea (99 [36.5%]), and neutropenia (96 [35.4%]) (Table 2). In the trastuzumab group, the most frequently reported TEAEs were alopecia (213 [76.6%] patients), neutropenia (116 [41.7%]), and nausea (94 [33.8%]). The proportions of patients experiencing study drug-related TEAEs were also similar between groups (Table S2 in Online Resource 1). In the CT-P6 group, the most frequently reported study drug-related TEAEs were infusion-related reactions (22 [8.1%] patients), alopecia (21 [7.7%]), and ejection fraction decreased (19 [7.0%]), while in the trastuzumab group these were neutropenia (30 [10.8%]), anemia (25 [9.0%]), and alopecia (24 [8.6%]). Treatment-emergent serious AEs (TESAEs) were reported for 21 (7.7%) and 35 (12.6%) patients in the CT-P6 and trastuzumab groups, respectively (Table 1). Similar proportions of patients experienced study drug-related TESAEs in each group (CT-P6: 6 [2.2%]; trastuzumab: 9 [3.2%]). Four deaths related to TEAEs occurred during the overall study, as previously reported [10, 11]. Two deaths occurred in the CT-P6 group during the neoadjuvant period and were associated with sudden death and dyspnea. In the trastuzumab group, 1 death due to acute myocardial infarction occurred during the neoadjuvant period; during the adjuvant period, there was 1 death due to aortic dissection.

**Table 1:** Safety findings during the overall study period (safety population)

	CT-P6 (*n* = 271)	Trastuzumab (*n* = 278)
Total number of TEAEs	2897	3136
Patients with ≥ 1 TEAE		
Any TEAE	263 (97.0)	265 (95.3)
Study drug–related	130 (48.0)	146 (52.5)
Total number of TESAEs	27	48
Patients with ≥ 1 TESAE		
Any TESAE	21 (7.7)	35 (12.6)
Study drug-related	6 (2.2)	9 (3.2)
Patients with cardiac disorders	32 (11.8)	39 (14.0)
Study drug-related	22 (8.1)	24 (8.6)

Data are *n* (%) of patients other than for the total number of TEAEs and TESAEs

*TEAE* treatment-emergent adverse event, *TESAE* treatment-emergent serious adverse event

**Table 2:** TEAEs reported for ≥ 10% of patients^a^ in either treatment group during the overall study period (safety population)

System organ class Preferred term	CT-P6 (*n* = 271)	Trastuzumab (*n* = 278)
Total number of TEAEs	2897	3136
Patients with ≥ 1 TEAE	263 (97.0)	265 (95.3)
Blood and lymphatic system disorders	132 (48.7)	155 (55.8)
Anemia	60 (22.1)	67 (24.1)
Leukopenia	28 (10.3)	40 (14.4)
Neutropenia	96 (35.4)	116 (41.7)
Cardiac disorders	32 (11.8)	39 (14.0)
Cardiac failure^a^	1 (0.4)	0
Cardiotoxicity^a^	1 (0.4)	1 (0.4)
Congestive cardiomyopathy^a^	0	1 (0.4)
Supraventricular tachycardia^a^	1 (0.4)	0
Gastrointestinal disorders	135 (49.8)	149 (53.6)
Diarrhea	52 (19.2)	50 (18.0)
Nausea	99 (36.5)	94 (33.8)
Stomatitis	46 (17.0)	33 (11.9)
Vomiting	27 (10.0)	26 (9.4)
General disorders and administration-site conditions	125 (46.1)	126 (45.3)
Asthenia	47 (17.3)	38 (13.7)
Fatigue	53 (19.6)	62 (22.3)
Pyrexia	31 (11.4)	30 (10.8)
Injury, poisoning, and procedural complications	75 (27.7)	76 (27.3)
Infusion-related reaction	31 (11.4)	29 (10.4)
Radiation skin injury	33 (12.2)	34 (12.2)
Investigations	68 (25.1)	69 (24.8)
Alanine aminotransferase increased	18 (6.6)	30 (10.8)
Ejection fraction abnormal^a^	0	1 (0.4)
Ejection fraction decreased^a^	20 (7.4)	9 (3.2)
Musculoskeletal and connective tissue disorders	76 (28.0)	86 (30.9)
Arthralgia	34 (12.5)	40 (14.4)
Myalgia	27 (10.0)	28 (10.1)
Skin and subcutaneous tissue disorders	210 (77.5)	224 (80.6)
Alopecia	196 (72.3)	213 (76.6)

Data are *n* (%) of patients other than for the total number of TEAEs

^a^TEAEs captured for heart failure under the system organ classes of cardiac disorders or investigations are presented regardless of frequency

*TEAE* treatment-emergent adverse event

During the follow-up period, similar proportions of patients in each treatment group experienced TEAEs (CT-P6: 8 [3.0%]; trastuzumab: 6 [2.2%]) (Table 3). Correspondingly, 4 (1.5%) and 3 (1.1%) patients experienced study drug-related TEAEs during the follow-up period. One (0.4%) and 2 (0.7%) patients in the CT-P6 and trastuzumab groups, respectively, experienced TESAEs during the follow-up period; TESAEs were related to study drug for 1 (0.4%) patient in each group (Table 3). The TESAE in the CT-P6 group was reported as Adams–Stokes syndrome. The investigator considered the event to be possibly related to study drug treatment despite the event occurring after the study drug treatment period and the patient’s medical history of transient second-degree atrioventricular block and hypertension. The study drug-related TESAE in the trastuzumab group was reported as dacryostenosis acquired, an eye disorder, which the investigator considered possibly related to study drug. The event was not newly reported during the post-treatment follow-up period: the patient experienced 2 non-serious occurrences of dacryostenosis acquired during the neoadjuvant period. No deaths due to TEAEs occurred during the follow-up period.

**Table 3:** Safety findings during the follow-up period (safety population)

	CT-P6 (*n* = 271)	Trastuzumab (*n* = 278)
Total number of TEAEs	9	6
Patients with ≥ 1 TEAE		
Any TEAE	8 (3.0)	6 (2.2)
Study drug-related	4 (1.5)	3 (1.1)
Total number of TESAEs	1	2
Patients with ≥ 1 TESAE		
Any TESAE	1 (0.4)	2 (0.7)
Study drug-related	1 (0.4)	1 (0.4)
Patients with cardiac disorders	3 (1.1)	3 (1.1)
Study drug-related	2 (0.7)	1 (0.4)

Data are *n* (%) of patients other than for total number of TEAEs and TESAEs

*TEAE* treatment-emergent adverse event, *TESAE* treatment-emergent serious adverse event

Overall, cardiac disorders were reported by 32 (11.8%) and 39 (14.0%) patients in the CT-P6 and trastuzumab groups, respectively (Table 1). Study drug-related cardiac disorders were experienced by 22 (8.1%) and 24 (8.6%) patients, correspondingly. TEAEs captured for heart failure are presented in Table 2. Within the system organ class *Cardiac disorders*, the incidence of most TEAEs was low and no imbalance was observed between treatment groups (Table S3 in Online Resource 1). The most frequent event was palpitations, reported in 10 (3.7%) and 8 (2.9%) patients in the CT-P6 and trastuzumab groups, respectively. During the follow-up period, 3 (1.1%) patients in each group experienced cardiac disorders (Table 4). Two (0.7%) patients in the CT-P6 group experienced cardiac disorders considered to be related to study drug: 1 case of Adams–Stokes syndrome (described previously) and 1 case of pericardial effusion. One (0.4%) patient in the trastuzumab group experienced a study drug-related cardiac disorder of mitral valve disease.

**Table 4:** TEAEs by system organ class reported during the follow-up period (safety population)

System organ class Preferred term	CT-P6 (*n* = 271)	Trastuzumab (*n* = 278)
Cardiac disorders	3 (1.1)	3 (1.1)
Adams–Stokes syndrome	1 (0.4)	0
Left ventricular hypertrophy	0	1 (0.4)
Mitral valve disease	0	1 (0.4)
Myocardial infarction	0	1 (0.4)
Palpitations	1 (0.4)	0
Pericardial effusion	1 (0.4)	0
Ventricular extrasystoles	1 (0.4)	0
Eye disorders	0	1 (0.4)
Dacryostenosis acquired	0	1 (0.4)
Gastrointestinal disorders	0	1 (0.4)
Abdominal pain	0	1 (0.4)
Infections and infestations	1 (0.4)	0
Nasopharyngitis	1 (0.4)	0
Investigations	3 (1.1)	1 (0.4)
Ejection fraction decreased	3 (1.1)	0
Electrocardiogram QT prolonged	0	1 (0.4)
Neoplasms benign, malignant, and unspecified (including cysts and polyps)	1 (0.4)	0
Breast adenoma	1 (0.4)	0

Data are *n* (%) of patients

*TEAE* treatment-emergent adverse event

As previously reported, median LVEF at baseline was 66.0% in both treatment groups (range, 55.0–83.0% [CT-P6 group] and 55.0–79.0% [trastuzumab group]) [10]. During the overall study period, most patients had a decrease of < 10 percentage points from baseline in LVEF (CT-P6: 169 [62.4%]; trastuzumab: 165 [59.4%]). Similar proportions of patients experienced significant LVEF decrease, defined as an absolute LVEF of < 50% with a decrease from baseline of ≥ 10 percentage points, in each treatment group (CT-P6: 9 [3.3%]; trastuzumab: 7 [2.5%]). As previously reported, 1 patient in the trastuzumab group exhibited symptoms of LVEF dysfunction and discontinued the study [10, 11]; the remaining patients had no signs or symptoms of LVEF dysfunction.

During the follow-up period, the median (range) worst post-baseline LVEF value was 61.0% (41.0–76.0%) in the CT-P6 group and 62.0% (43.3–79.0%) in the trastuzumab group. The majority of patients had an increase, no change, or a decrease of < 10 percentage points from baseline in LVEF (CT-P6: 204 [75.3%]; trastuzumab: 208 [74.8%]). Three (1.1%) and two (0.7%) patients in the CT-P6 and trastuzumab groups, respectively, experienced significant LVEF decrease, and 3 (1.1%) patients in the CT-P6 group only reported TEAEs under the Preferred Term *ejection fraction decreased* within system organ class *Investigations* (Table 4). LVEF recovered in all cases and none of these patients had signs or symptoms of LVEF dysfunction.

As previously reported, all postinfusion ADA results were negative during the neoadjuvant and adjuvant periods [10, 11]. During the post-treatment follow-up, 2 patients in the CT-P6 group were ADA positive; both patients tested negative for neutralizing antibodies.

## Discussion

Most patients with operable HER2-positive early breast cancer who were enrolled in this study entered the follow-up period. Therefore, this study provides robust data from the post-treatment setting after neoadjuvant and adjuvant treatment with either CT-P6 or trastuzumab. The similarity of the time-to-event analyses between treatment groups provides long-term data to support the conclusion of equivalence of CT-P6 and trastuzumab drawn for the primary endpoint [10]. In addition, CT-P6 was well tolerated, with a comparable safety profile to that of trastuzumab throughout the study.

In terms of time-to-event analyses, our findings were comparable with those of previous studies that had evaluated the treatment of early or operable HER2-positive breast cancer with trastuzumab administered in both the neoadjuvant and adjuvant settings. The 3-year OS identified in our study (CT-P6: 0.93; trastuzumab: 0.94) is comparable to findings from the phase III HannaH study, in which the 3-year OS rate was 0.90 for patients receiving neoadjuvant and adjuvant therapy, including intravenous trastuzumab [12], and to the OS of 0.90 demonstrated with neoadjuvant and adjuvant trastuzumab therapy in the phase III NeoALTTO study [13]. Our findings were also broadly comparable to the 3-year OS of 0.98 reported in the phase II JBCRG-10 study, which evaluated different sequences of trastuzumab-containing therapy in the neoadjuvant setting [14], and the 3-year OS of 0.87 in a retrospective study that evaluated a trastuzumab-containing neoadjuvant regimen, followed by up to 1 year of trastuzumab [15].

In terms of DFS, the 3-year rates of 0.83 for both groups in the present study were similar to the 3-year rate (0.84) in a retrospective study of patients treated with a trastuzumab-containing neoadjuvant regimen, followed by up to 1 year of trastuzumab [15]. DFS findings were also broadly comparable to 3-year results from 2 studies evaluating trastuzumab-containing therapy in the neoadjuvant setting only (0.97 in the JBCRG-10 study [14] and 0.95 in a retrospective and prospective observational study [16]). Our DFS findings were in line with the 3-year rates of 0.87 and 0.89 for patients with HER2-positive early breast cancer treated with trastuzumab for 1 or 2 years, respectively, in the phase III HERA trial, which enrolled patients who had undergone diverse primary treatment (including surgery, neoadjuvant chemotherapy, adjuvant chemotherapy, and radiation therapy) [17].

To our knowledge, PFS data for comparable treatment regimens and patient populations are not available. However, our 3-year PFS rates (CT-P6: 0.81; trastuzumab: 0.87) are similar to the 3-year event-free survival rates of 0.76 reported following neoadjuvant and adjuvant trastuzumab therapy in the NeoALTTO study [13] and 0.73 reported for the intravenous trastuzumab arm in the HannaH study [12].

The overall long-term safety findings of the present study were consistent with the known safety profile of trastuzumab [4, 5] and there were no new or unexpected safety findings. One important element of the trastuzumab safety profile is cardiac safety [18]. In our study, 11.8% and 14.0% of patients in the CT-P6 and trastuzumab groups, respectively, experienced cardiac disorders. This is comparable to the 13.4% of intravenous trastuzumab-treated patients with HER2-positive early breast cancer reporting cardiac events during the study period (which included 2 years of treatment-free follow-up) in the HannaH study [12], and the 8.3% of patients receiving subcutaneous or intravenous trastuzumab-containing regimens who had experienced cardiac AEs in the phase II PrefHer study [19]. In keeping with the rare occurrence of cardiac events after completion of trastuzumab treatment in the HERA trial [17] and the 1.3% incidence of cardiac AEs for intravenous trastuzumab-treated patients during treatment-free follow-up in the HannaH study [12], few patients (3 [1.1%] in each group) reported cardiac disorders during the follow-up period of our study, while no longer receiving trastuzumab. TEAEs captured for heart failure were uncommon, with relevant Preferred Terms reported by at most 1 (0.4%) patient in each group, other than for ejection fraction decreased (reported by 20 [7.4%] and 9 [3.2%] patients in the CT-P6 and trastuzumab groups, respectively, overall). The incidence of ejection fraction decreased was in line with expectations based on information for the reference product from clinical trial and post-marketing settings (frequency ≥ 1/10), while the incidence of other TEAEs captured for cardiac failure was in keeping with the expected frequency for comparable terms listed in the reference product information (≥ 1/100 to < 1/10) [5].

The interpretation of our findings is limited by the trial not being powered for survival, as previously noted [10, 11]. Conclusions about long-term efficacy are also limited by the relatively short follow-up (median of 38.7 and 39.6 months in the CT-P6 and trastuzumab groups, respectively). However, the CT-P6 4.2 extension study (EudraCT number: 2019-003518-15) is ongoing. The CT-P6 4.2 study will collect data for up to 3 years after the last follow-up visit in the CT-P6 3.2 study, enabling analysis of 6-year DFS, PFS, and OS data.

The availability of biosimilars can increase the number of treatment options for both clinicians and patients. Reduced prices for biosimilars, compared with reference products, may improve access to biologic therapies, helping to improve financial sustainability of healthcare systems in the face of increasing drug costs in oncology [20]. For CT-P6, in particular, a budget impact analysis has considered the effect of switching from trastuzumab to CT-P6 for the treatment of early breast cancer, metastatic breast cancer, and metastatic gastric cancer in 28 European countries, and predicted substantial cost savings that could improve patient access to biologic treatment [21].

In summary, the similarity of DFS, PFS, and OS between CT-P6 and trastuzumab, demonstrated in this analysis after up to 3 years’ follow-up, supports the conclusion of equivalence in terms of efficacy previously established between the biosimilar and reference product. In addition, CT-P6 was well tolerated, with comparable safety and immunogenicity profiles to trastuzumab.

## Supplementary Information

Below is the link to the electronic supplementary material.

Supplementary file1 (DOCX 887 kb)

## Data Availability

Any requests for deidentified data and supporting materials will be considered for qualified external researchers who provide a methodologically sound proposal. Proposals should be directed to the corresponding author in the first instance. These requests are reviewed and approved by Celltrion, Inc. (Incheon, Republic of Korea), on the basis of scientific merit. All data provided are anonymized to respect the privacy of patients who have participated in the trial, in line with applicable laws and regulations.
