# Supplementary material for: Long-term efficacy and safety of CT-P6 versus trastuzumab in patients with HER2-positive early breast cancer: final results from a randomized phase III trial
Source: Breast Cancer Res Treat. 2021 Jun 20;188(3):631–40. doi: 10.1007/s10549-021-06240-5 (PMC8272708; doi:10.1007/s10549-021-06240-5)
Supplement: Supplementary file 1 — Supplementary file1 (DOCX 887 kb) [file 10549_2021_6240_MOESM1_ESM.docx]

## Online Resource 1

**Fig. S1** Study design. ^a^Each cycle lasted 3 weeks. ^b^Up to 3 years from the date of enrollment of the last patient. ^c^In place of the usual 6 mg/kg dose, an 8 mg/kg loading dose was administered on day 1 of cycle 1 in the neoadjuvant period only. Figure adapted from Esteva FJ, et al (2019), Efficacy and safety of CT-P6 versus reference trastuzumab in HER2-positive early breast cancer: updated results of a randomised phase 3 trial. Cancer Chemother Pharmacol 84:839–847, as permitted under the terms of the Creative Commons Attribution 4.0 International License (http://creativecommons.org/licenses/by/4.0/)


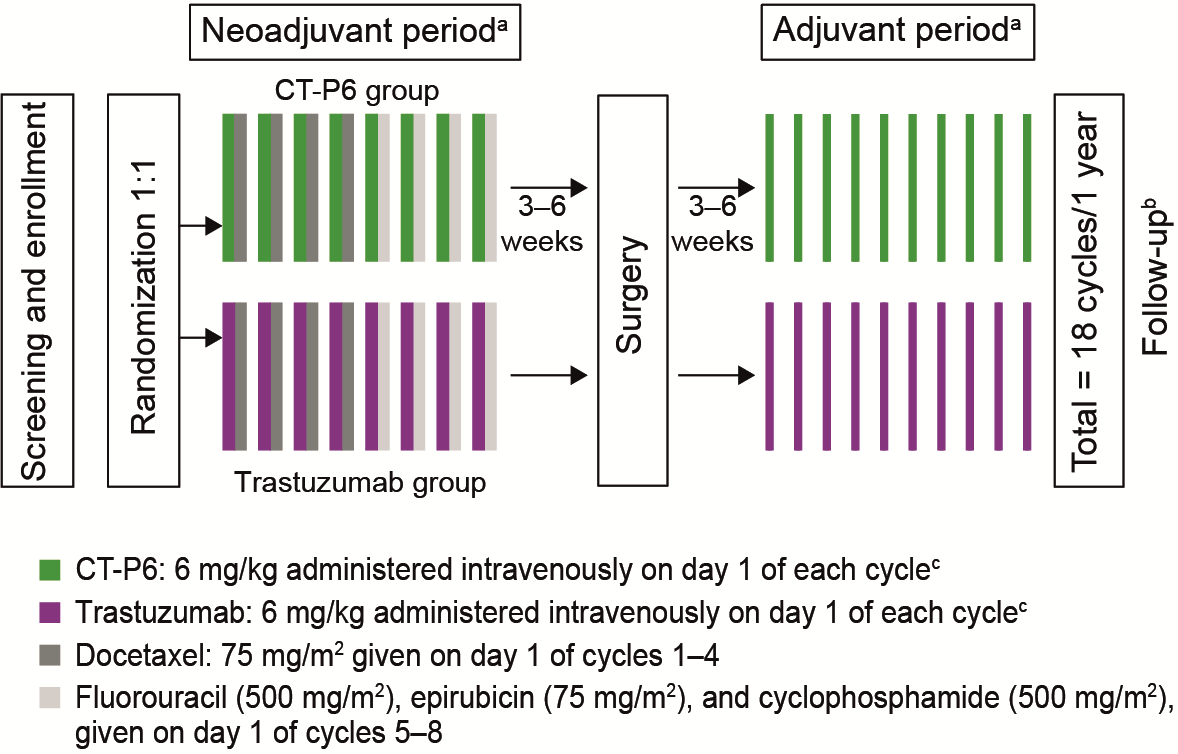


**Table S1** Progressive disease/recurrence events for disease-free survival (intention-to-treat population)

|  | **CT-P6**  **(*n*=258)** | **Trastuzumab**  **(*n*=261)** |
| --- | --- | --- |
| Progressive disease/recurrence | 41 (15.9) | 33 (12.6) |
| Distant | 25 (9.7) | 25 (9.6) |
| Locoregional | 14 (5.4) | 7 (2.7) |
| Other | 2 (0.8)^a^ | 1 (0.4)^b^ |

Data are *n* (%) of patients

^a^Progression at post-surgical suture area (*n*=1); further information not available (*n*=1)

^b^Data not available due to assessment at a different hospital

**Fig. S2** Kaplan–Meier plots for time-to-event analyses (per-protocol population). **a** Follow-up duration. **b** Disease-free survival. **c** Progression-free survival. **d** Overall survival. *CI* confidence interval, *HR* hazard ratio


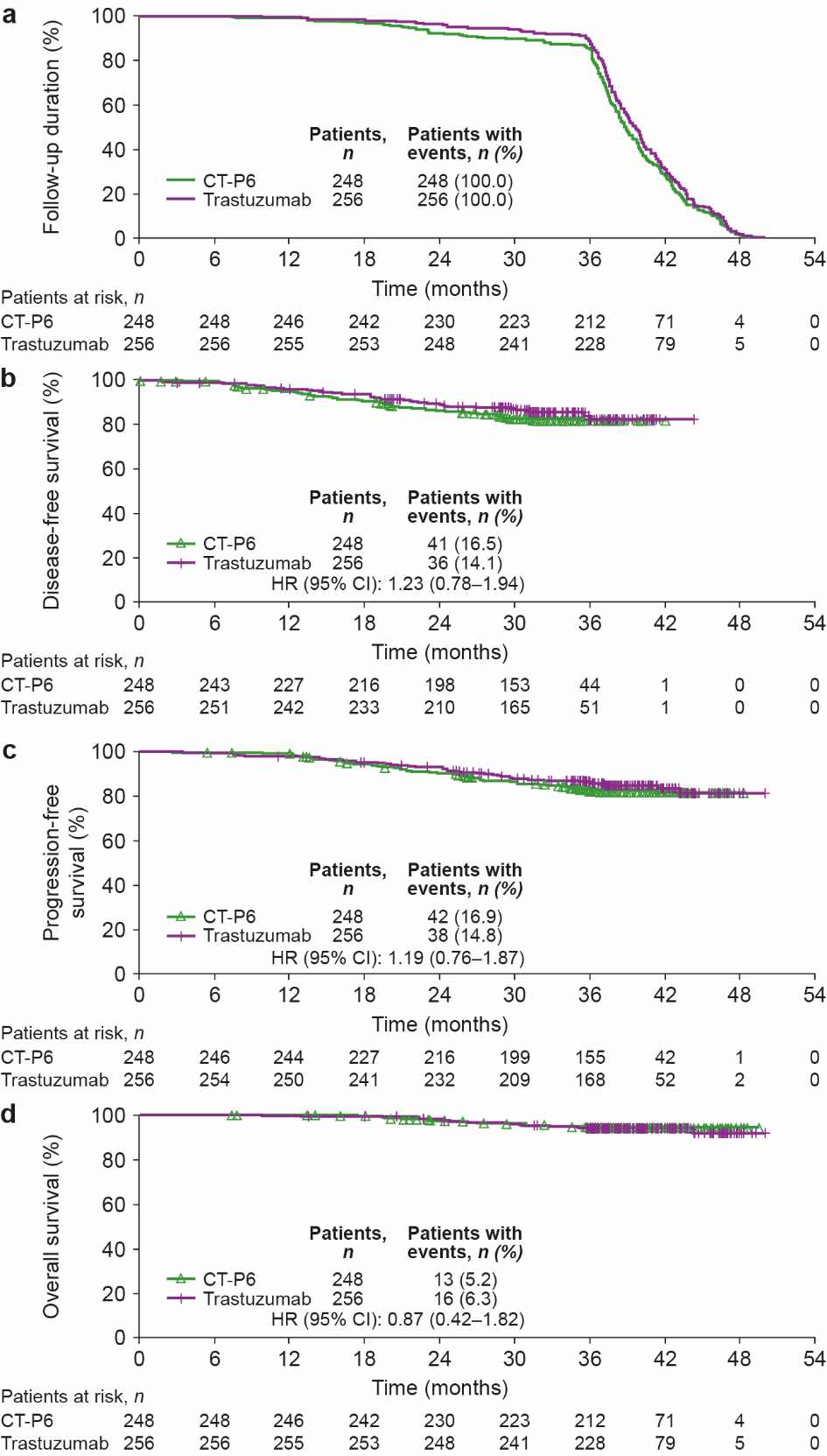


**Table S2** Study drug–related TEAEs reported for ≥3% of patients in either treatment group during the overall study period (safety population)

| **System Organ Class**  **Preferred Term** | **CT-P6**  **(*n*=271)** | **Trastuzumab**  **(*n*=278)** |
| --- | --- | --- |
| Patients with ≥1 TEAE | 130 (48.0) | 146 (52.5) |
| Blood and lymphatic system disorders | 24 (8.9) | 46 (16.5) |
| Anemia | 11 (4.1) | 25 (9.0) |
| Leukopenia | 6 (2.2) | 14 (5.0) |
| Neutropenia | 16 (5.9) | 30 (10.8) |
| Eye disorders | 17 (6.3) | 15 (5.4) |
| Lacrimation increased | 13 (4.8) | 8 (2.9) |
| Gastrointestinal disorders | 35 (12.9) | 33 (11.9) |
| Constipation | 8 (3.0) | 4 (1.4) |
| Diarrhea | 14 (5.2) | 12 (4.3) |
| Nausea | 15 (5.5) | 20 (7.2) |
| Stomatitis | 8 (3.0) | 7 (2.5) |
| General disorders and administration-site conditions | 40 (14.8) | 39 (14.0) |
| Asthenia | 11 (4.1) | 6 (2.2) |
| Fatigue | 13 (4.8) | 17 (6.1) |
| Injury, poisoning, and procedural complications | 22 (8.1) | 18 (6.5) |
| Infusion-related reaction | 22 (8.1) | 18 (6.5) |
| Investigations | 30 (11.1) | 41 (14.7) |
| Alanine aminotransferase increased | 4 (1.5) | 16 (5.8) |
| Aspartate aminotransferase increased | 2 (0.7) | 15 (5.4) |
| Ejection fraction decreased | 19 (7.0) | 8 (2.9) |
| Musculoskeletal and connective tissue disorders | 17 (6.3) | 18 (6.5) |
| Arthralgia | 5 (1.8) | 13 (4.7) |
| Nervous system disorders | 14 (5.2) | 17 (6.1) |
| Headache | 10 (3.7) | 3 (1.1) |
| Peripheral sensory neuropathy | 2 (0.7) | 9 (3.2) |
| Skin and subcutaneous tissue disorders | 57 (21.0) | 51 (18.3) |
| Alopecia | 21 (7.7) | 24 (8.6) |
| Rash | 10 (3.7) | 5 (1.8) |

Data are *n* (%) of patients

*TEAE* treatment-emergent adverse event

## Table S3 TEAEs reported under System Organ Class *Cardiac disorders* during the overall study period (safety population)

| **System Organ Class**  **Preferred Term** | **CT-P6**  **(*n*=271)** | **Trastuzumab**  **(*n*=278)** |
| --- | --- | --- |
| Cardiac disorders | 32 (11.8) | 39 (14.0) |
| Acute myocardial infarction | 0 | 1 (0.4) |
| Adams–Stokes syndrome | 1 (0.4) | 0 |
| Angina pectoris | 2 (0.7) | 1 (0.4) |
| Aortic valve disease | 0 | 1 (0.4) |
| Aortic valve incompetence | 0 | 1 (0.4) |
| Arteriosclerosis coronary artery | 0 | 1 (0.4) |
| Bundle branch block left | 0 | 1 (0.4) |
| Bundle branch block right | 1 (0.4) | 0 |
| Cardiac disorder | 0 | 1 (0.4) |
| Cardiac failure | 1 (0.4) | 0 |
| Cardiac hypertrophy | 1 (0.4) | 0 |
| Cardiomyopathy | 1 (0.4) | 5 (1.8) |
| Cardiotoxicity | 1 (0.4) | 1 (0.4) |
| Congestive cardiomyopathy | 0 | 1 (0.4) |
| Coronary artery disease | 2 (0.7) | 0 |
| Diastolic dysfunction | 0 | 1 (0.4) |
| Dilatation atrial | 0 | 1 (0.4) |
| Dilatation ventricular | 0 | 1 (0.4) |
| Extrasystoles | 1 (0.4) | 1 (0.4) |
| Hypertensive heart disease | 0 | 1 (0.4) |
| Left atrial hypertrophy | 0 | 1 (0.4) |
| Left ventricular dysfunction | 0 | 1 (0.4) |
| Left ventricular hypertrophy | 0 | 2 (0.7) |
| Metabolic cardiomyopathy | 0 | 1 (0.4) |
| Mitral valve disease | 0 | 2 (0.7) |
| Mitral valve incompetence | 3 (1.1) | 4 (1.4) |
| Mitral valve prolapse | 0 | 1 (0.4) |
| Myocardial infarction | 0 | 1 (0.4) |
| Myocardial ischemia | 1 (0.4) | 0 |
| Palpitations | 10 (3.7) | 8 (2.9) |
| Pericardial effusion | 1 (0.4) | 2 (0.7) |
| Pericarditis | 2 (0.7) | 1 (0.4) |
| Right ventricular failure | 0 | 1 (0.4) |
| Sinus tachycardia | 2 (0.7) | 3 (1.1) |
| Supraventricular extrasystoles | 1 (0.4) | 1 (0.4) |
| Supraventricular tachycardia | 1 (0.4) | 0 |
| Tachyarrhythmia | 0 | 1 (0.4) |
| Tachycardia | 6 (2.2) | 5 (1.8) |
| Tricuspid valve incompetence | 2 (0.7) | 0 |
| Ventricular extrasystoles | 1 (0.4) | 0 |
| Ventricular hyperkinesia | 1 (0.4) | 1 (0.4) |

Data are *n* (%) of patients

*TEAE* treatment-emergent adverse event
